# Synergy Between Two Chimeric Lysins to Kill *Streptococcus pneumoniae*

**DOI:** 10.3389/fmicb.2019.01251

**Published:** 2019-06-05

**Authors:** Roberto Vázquez, Pedro García

**Affiliations:** ^1^Departamento de Biotecnología Microbiana y de Plantas, Centro de Investigaciones Biológicas, CSIC, Madrid, Spain; ^2^CIBER de Enfermedades Respiratorias, Madrid, Spain

**Keywords:** pneumococcus, lysin, synergy, biofilm, zebrafish model

## Abstract

Phage lysins constitute a new generation of antimicrobials that are becoming a promising alternative and complementation to current antibiotic therapies, which are nowadays called into question by the increasing numbers of multiresistant bacteria. *Streptococcus pneumoniae* is a leading human pathogen causing serious infectious diseases in children and adults. Within the host-parasite interplay system of pneumococcus and its phages, several antipneumococcal lysins have been described and, among them, chimeric lysins Cpl-711 and PL3 stand out for their potent bactericidal activities. Here, evidence is presented on the synergistic cooperation of the catalytically diverse lysins Cpl-711 and PL3 in different assays, like purified cell wall enzymatic degradation, *in vitro* bacterial cell growth inhibition, and killing of both planktonic and biofilm grown cells. Synergy between Cpl-711 and PL3 has been shown to reduce the amount of enzyme necessary to inhibit growth in checkerboard assays with a sum of fractional inhibitory concentrations ≤0.5 for all pneumococcal strains tested, while also significatively increasing bactericidal effect by ≥2 logs with respect to the sum of activities of Cpl-711 and PL3 individual treatments. Moreover, the combination of these two lysins showed synergy in an adult zebrafish model of pneumococcal infection. This study consolidates the possibility of formulating highly efficient and synergistic antibacterial enzymes that could improve our ability to fight multiresistant bacterial infections.

## Introduction

Interest in developing novel ways of treating infectious diseases is currently growing due to the worrisome reports on the increase of multidrug resistant (MDR) bacterial strains (Tacconelli et al., 2018), which threatens to hinder our ability to successfully treat bacterial infections with our current antibiotic-based therapies. These so-called “superbugs” seem an outcome of the selective pressure exerted by human use and abuse of antibiotics, not only in human healthcare settings but also in farming and food production industries (O'Neill, 2016). Prompted by several institutional calls (O'Neill, 2016; Tacconelli et al., 2018), researchers and institutions have set an aim in devising novel strategies to fight bacterial infections. One of such novel strategies are phage-derived lysins (Pastagia et al., 2013). Phage lysins (also referred to as “endolysins” or “enzybiotics”) are enzymes that break specific bonds of bacterial peptidoglycan and allow the release of mature virion particles from host cells leading to cell lysis and death. Currently, these enzymes are being repurposed for bacterial killing from outside the cell. In this way, lysins gain access to its target and degrade the peptidoglycan causing cell death by osmotic shock. Phage lysins represent a new generation of antimicrobials that have several advantages over conventional antibiotics: (1) they are very fast in their lethal activity, practically upon contact with the bacteria; (2) in general, they are very specific to the pathogen, which allows to preserve the normal microbiota; (3) the appearance of resistant mutants seems very unlikely, probably because the peptidoglycan is an essential and well-conserved structural component that cannot be easily modified without compromising their fitness (Pastagia et al., 2013, and the references therein). The intrinsic killing activity of phage lysins can even be improved by engineering the wild type enzymes (São-José, 2018) and/or combining them with other lysins, antibiotics or certain compounds to get a synergistic effect (Letrado et al., 2018).

*Streptococcus pneumoniae* is an important human pathogen, responsible for different kinds of infections (e.g., pneumonia, otitis, meningitis, sepsis), and the most frequent causative agent of community-acquired pneumonia (Rodrigues and Groves, 2018). According to global health estimates, lower respiratory tract infections remain the most deadly communicable diseases (World Health Organization, 2017), with *S. pneumoniae* as the leading causative pathogen (Rodrigues and Groves, 2018). While *S. pneumoniae* is considered a vaccine-preventable pathogen, the multivalent vaccines currently in use, based on capsular antigens, have the important drawbacks of serotype replacement (Weinberger et al., 2011) and defective protection against non-encapsulated pneumococci, which are starting to increase in prevalence (Keller et al., 2016). Moreover, the high percentage of pneumococcal isolates resistant to β-lactams and/or macrolides (Cherazard et al., 2017) has made *S. pneumoniae* enter the WHO priority list for novel antibiotics development (Tacconelli et al., 2018). Thus, *S. pneumoniae* has also been the subject of several lysin-based approaches for devising new therapies (Vázquez et al., 2018). The peptidoglycan-degrading enzymes of the pneumococcal system have been extensively studied (López and García, 2004). All of them display a modular architecture, a feature shared by many phage lysins from Gram-positive bacteria and, with the exception of the Cpl-7 lysin from phage Cp-7 (Bustamante et al., 2010), all belong to the choline-binding proteins (CBP) family. This means that, besides a catalytic domain (either a 1,4-*N*-acetylmuramidase activity [muramidase or lysozyme] or an *N*-acetylmuramoyl-L-alanine amidase activity [NAM-amidase]) these enzymes contain a cell wall-binding domain (CWBD) that specifically recognizes choline residues in the teichoic acids of *S. pneumoniae* and a few other related bacteria, thus being named choline binding domains (CBDs). Several CBD-containing lysins have been demonstrated to act as effective weapons to specifically kill pneumococci, both susceptible and MDR strains (Loeffler et al., 2001; Jado et al., 2003; Rodríguez-Cerrato et al., 2007b; Domenech et al., 2011). Lethal effect of some of these lysins has been improved by means of modular protein engineering, rendering the chimeric protein Cpl-711, which contains the catalytic module of Cpl-7 and the CBD of Cpl-1 (Díez-Martínez et al., 2015); and PL3, comprising the catalytic module of Pal and part of the CBD of LytA (Blázquez et al., 2016). These chimeras, Cpl-711 and PL3, showed an increased bactericidal and therapeutic activity with respect to their parental enzymes (Díez-Martínez et al., 2013; Blázquez et al., 2016; Corsini et al., 2018). It is noteworthy that antipneumococcal lysins have demonstrated synergy with a variety of antibiotics (Rodríguez-Cerrato et al., 2007a; Vouillamoz et al., 2013; Letrado et al., 2018), and also displayed a synergistic effect when they catalyze the breakage of different peptidoglycan bonds. This is the case of Cpl-1 lysozyme and Pal or LytA NAM-amidases (Jado et al., 2003; Loeffler and Fischetti, 2003; Rodríguez-Cerrato et al., 2007a; Domenech et al., 2011).

In this study, we have tested the combination of Cpl-711 and PL3 as the most active examples of lysozyme and NAM-amidase activities against pneumococci, both in planktonic culture and in biofilm-growing conditions. These *in vitro* results have been validated in a zebrafish model of infection. A synergistic effect between these two lysins, which greatly reduces the amount of enzymes necessary to achieve high bactericidal and therapeutic effects, has been demonstrated.

## Materials and Methods

### Bacterial Strains and Plasmids

The pneumococcal strains employed throughout this work are detailed in Table 1. All *S*. *pneumoniae* strains were routinely grown in C medium supplemented with yeast extract (0.8 mg/ml) (C + Y medium) (Lacks and Hotchkiss, 1960) at 37°C without shaking. *Staphylococcus aureus* CECT 86 was cultured in Brain Heart Infusion (BHI) broth at 37°C without shaking. All antipneumococcal lysins were expressed in *Escherichia coli* strains (grown in LB medium at 37°C with shaking). The strains and plasmids used are shown in Table S1.

**Table 1:** Pneumococcal strains used in this study.

**Strain**	**Serotype**	**Observations^**a**^**	**References**
ATCC 49619	19F	Quality control for MIC determination experiments	ATCC^b^
R6	Non-encapsulated	Usual workhorse strain for *in vitro S. pneumoniae* experiments	Hoskins et al., 2001
D39	2	Parental strain of R6	Lanie et al., 2007
P042	Non-encapsulated	R6 *lytA lytC* mutant. Used for avoiding autolysins interference when assessing the effect of lytic enzymes	Moscoso et al., 2006
48	23F	MDR strain (AMX MIC = 16 μg/ml; ERY MIC = 1,024 μg/ml; CTX MIC = 16 μg/ml; TET MIC = 128 μg/ml)	Bustamante et al., 2010
69	19F	MDR strain (PEN MIC = 2 μg/ml; CRO MIC = 2 μg/ml; CDN MIC = 2 μg/ml)	Ramos-Sevillano et al., 2012

a *AMX, amoxicillin; ERY, erythromycin; CTX, cefotaxime; TET, tetracycline; PEN, penicillin; CRO, ceftriaxone; CDN, cefditoren*.

b *ATCC, American Type Culture Collection*.

### Protein Expression and Purification

Lysins (LytA, Pal, PL3, Cpl-7, Cpl-1, and Cpl-711) were expressed in 2 liter cultures of *E. coli* cells supplemented with the corresponding antibiotics and adding 0.1–0.4 mM isopropyl-β-D-thiogalactopyranoside upon reaching optical density at 550 nm (OD_550_) ≈ 0.6. After an incubation period at the previously described conditions for each protein (further details can be found in the references included in Table S1), cultures were disrupted with a French press, DNA was precipitated with streptomycin sulfate and proteins were subsequently purified from supernatants. All choline-binding lysins (Cpl-1, Cpl-711, LytA, and PL3) were purified in a single step by DEAE-Sepharose affinity chromatography as described elsewhere (Sánchez-Puelles et al., 1990). Figure S1 depicts an SDS-PAGE showing expression and purification of Cpl-711 and PL3. Cpl-7 was purified via a two-step protocol involving fractional precipitation with ammonium sulfate and DEAE-Sepharose anionic exchange chromatography with a NaCl elution gradient (Bustamante et al., 2010). All chromatographic steps were performed using a HiTrap DEAE-Sepharose FF 5 ml column (GE Healthcare). Protein concentrations were determined spectrophotometrically using the theoretical molar absorption coefficient at 280 nm calculated using the ProtParam program.

### *In vitro* Cell Wall Lytic Activity Assay

The cell wall degradation activity of the selected lysins and their putatively synergistic combination was tested on purified R6 pneumococcal cell walls, prepared as described elsewhere (Garcia-Bustos and Tomasz, 1987). Cell walls were labeled with Remazol Brilliant Blue (RBB, Sigma), as previously described (Zhou et al., 1988). In short, purified pneumococcal cell walls were centrifuged and resuspended in a freshly prepared 0.25 M NaOH solution containing 20 mM RBB using 60 ml/g of cell walls. The suspension was incubated for 6 h at 37°C with shaking, and then for 12 h at 4°C. The stained cell walls were repeatedly washed with water by centrifugation until the supernatant was colorless. To measure cell wall solubilization by the catalytic activities tested, 150 μl of an OD_595_ ≈ 0.5 suspension of RBB-stained cell walls were pelleted and resuspended in 150 μl of a solution containing 1.3μM (≈ 50 μg/ml) of either PL3, Cpl-711 or a 1:10 mixture of both (MIX) in 20 mM sodium phosphate, pH 6.5, 150 mM NaCl, 10 mM dithiothreitol buffer (PB) or just PB as a no-treatment control and spectrophotometric blank read. Incubation was performed at 37°C and, after centrifugation (16,000 × *g*, 10 min), solubilized degradation products were measured from 100 μl samples of the supernatant as OD_595_ using a VersaMax microplate absorbance reader (Molecular Devices).

### Minimal Inhibitory Concentration (MIC) and *in vitro* Synergy Determination

MICs were determined according to the Clinical and Laboratory Standards Institute (CSLI) guidelines for *S. pneumoniae* antimicrobial susceptibility testing (CLSI, 2018). Briefly, 5 × 10^5^-10^6^ CFU/ml were plated onto each well of a 96-well microplate containing 2-fold dilutions of the filter-sterilized enzymes in cation-adjusted Mueller-Hinton broth supplemented with 5% lysed horse blood (CAMH+LHB). A 2-fold dilution scheme was followed, ranging from 100 to 0.10 μg/ml of each compound. Plates were incubated at 37°C for 20–24 h in a 5% CO_2_ atmosphere and examined to visually confirm either growth or not. Interactions between two enzymes were tested by the checkerboard assay (CLSI, 1999). Such assays were performed in the same microtiter plates using the same medium and incubation conditions as described for MIC determinations. Compounds were usually tested in a range from 1/32 to 2× MIC. The fractional inhibitory concentration (FIC) for each well was calculated (FIC_A_ = C_A_/MIC_A_; where FIC_A_ is the fractional inhibitory concentration of compound A, C_A_ is the concentration of compound A in a given well and MIC_A_ is the MIC of compound A) and the sum of FIC or FIC index (FICI = FIC_A_ + FIC_B_) was used to assess whether the combination was synergistic. Synergy was defined, as usual, by a FICI ≤ 0.5, antagonism by a FICI > 4.0 and no interaction (or mere additive interaction) by a FICI of 0.5–4.0. Checkerboard assays were complemented by time-kill curves with the selected combinations of lysins (CLSI, 1999). These assays were performed in CAMH+LHB medium with each compound alone or in combination at 0.25× MIC. Bacteria were grown at 37°C to stationary phase at a final concentration of ≈ 10^8^ CFU/ml, taking samples after 2, 4 and 7 h of incubation. As in previous works (Rodríguez-Cerrato et al., 2007a; Letrado et al., 2018), incubation time was no further prolonged due to *S. pneumoniae* autolysis. Serial dilutions of samples were cultured onto blood agar plates for viable cell titer determination. Each test was performed at least three separate times.

### *In vitro* Biofilm Assays

*S. pneumoniae* P042 biofilms were prepared as described elsewhere (Domenech et al., 2011). Briefly, *S. pneumoniae* P042 strain was grown in C+Y medium to OD_550_ ≈ 0.6 and diluted 1/2,000 in C medium. Then, 200 μl (≈ 10^4^-10^5^ CFU) were plated per well in Costar 3595 96-well polystyrene microtiter plates. After 14–16 h incubation at 34°C, antibiofilm treatment was applied by removing the non-adherent bacteria and replacing it with PB containing the respective lysins or combinations. Incubation was therefore continued for an additional hour at 37°C. Viable cells were determined both in the planktonic fraction and the adhered biofilm, in the latter case resuspending adhered cells in 100 μl of the same buffer solution with a previous washing step.

### Zebrafish Model Synergy Assays

Zebrafish embryos acute toxicity assay was performed by exposing 0–72 h post fecundation embryos (N = 12 per condition) by immersion to 200 μl solutions of different concentrations of Cpl-711/PL3 mixtures (0.5× to 2× MIC of each enzyme). Both a positive control (1 mg/ml paracetamol) and a negative control (E3 medium) were included. Embryos were incubated at 28°C for 72 h and observed for external signs of toxicity (coagulated embryos, delay in somite formation, spine deformation, slow heartbeat, hepatic necrosis, oedema, etc.). Signs of infection were monitored three times daily throughout the experimental time course. To determine *in vivo* antimicrobial synergy, an adult zebrafish infection model (Letrado et al., 2018) was used. Zebrafish (6 per condition and replicate, with three replicates, *N* = 18) were infected with 10 μl of an *S. pneumoniae* strain 48 suspension (≈ 10^6^ CFU/ml) by the intraperitoneal route. One hour after infection, zebrafish were divided into four groups and treated intraperitoneally with 10 μl of subtherapeutic doses of Cpl-711 (0.5 μg/ml) or PL3 (0.15 μg/ml), or with a combination of Cpl-711 and PL3 at half the previous doses (0.25 μg/ml Cpl-711 plus 0.08 μg/ml PL3), or with 20 mM sodium phosphate, 150 mM NaCl, pH 6.0 buffer, as a control. Therapeutic doses were, then, for an estimated zebrafish weight of 1 g: 5.00 mg/kg Cpl-711, 1.50 mg/kg PL3 and a mixture of 2.50 mg/kg Cpl-711 plus 0.75 mg/kg PL3. The survival rate for each experimental group was monitored every 24 h for up to 3 days after the infection.

### Video Microscopy

Exponential cultures of either the pneumococcal P042 strain alone (Video S1), or with *S. aureus* CECT 86 strain (mixed at the same OD_550_ ≈ 0.3) (Video S2), were centrifuged and the pellet washed twice with distilled water. The cells were then resuspended in distilled water and adjusted to OD_550_ ≈ 0.6. A sample of both bacterial suspensions was spread onto a microscope slide and let air-dry. Finally, 5 μl of a solution containing the final concentration of Cpl-711 and PL3 (10× MIC) were added, and observed with a Leica DM4000B microscope under a 100× oil objective. Recording started immediately after the enzyme addition, using an iPhone 6S with an adapter for mounting it onto the ocular lens. Videos are shown in real time.

### Statistical Analysis

All *in vitro* results are data obtained from at least three repeated independent experiments, showing in all cases the mean ± standard deviation unless otherwise stated. Statistical analysis was performed by analysis of variance (ANOVA) followed by either Tukey or Bonferroni post-tests, or *t*-Student tests for mean comparisons when indicated. For *in vivo* data, the log-rank (Mantel–Cox) and Gehan–Breslow–Wilcoxon tests were used to draw, analyze and compare the survival curves. GraphPad InStat version 5.0 (GraphPad Software, San Diego, CA) was used for all the analysis.

## Results

### MICs of Antipneumococcal Lysins

MICs of relevant lysins used to kill pneumococci, including Cpl-711 and PL3 and their parental enzymes, were determined for the non-encapsulated strain R6, its *lytA lytC* derivative P042, and the encapsulated parental strain D39, as well as the clinical MDR strains 48 and 69, and the CLSI-recommended quality control strain ATCC 49619 (Table 1). PL3 had the lowest MIC value among all the lysins tested here (0.10–0.50 μg/ml, corresponding to 3–15 nM), about one order of magnitude lower than that of Cpl-711 for any strain (0.80–4.61 μg/ml, corresponding to 20–115 nM) (Table 2). The MICs of these two chimeric enzymes (Cpl-711 and PL3) were also substantially lower than those of their corresponding most potent parental proteins (Cpl-1 and LytA for Cpl-711 and PL3, respectively).

**Table 2:** MICs (μg/ml) for each lysin used in this work determined for several *S. pneumoniae* strains^a^.

	**ATCC 49619**	**P042**	**R6**	**D39**	**48**	**69**
Cpl-7	>100	19.8	21.0	19.8	19.8	>100
Cpl-1	17.7	4.0	4.3	3.1	4.5	8.2
Pal	>100	3.1	9.9	3.9	63.0	50
LytA	0.6	0.3	0.4	0.2	0.4	0.4
Cpl-711	4.6	1.0	1.3	0.8	1.3	4.0
PL3	0.5	0.2	0.1	0.2	0.3	0.2
Cpl-1 vs. Cpl-711^b^	***	***	***	***	***	ns
LytA vs. PL3^b^	ns	*	**	ns	ns	ns

a *Data are the geometric mean of at least three independent MIC experiments*.

b Statistical significance of comparisons between MICs of chimeric lysins and their most potent parental enzyme according to two-way ANOVA followed by Bonferroni post-test performed on the log_2_-transformed MIC values is shown below the table (*P < 0.05; ^**^P < 0.01; ^***^P < 0.001; ns = non-significant difference).

### Synergistic Effect of Cpl-711 and PL3

**Figure 1:**
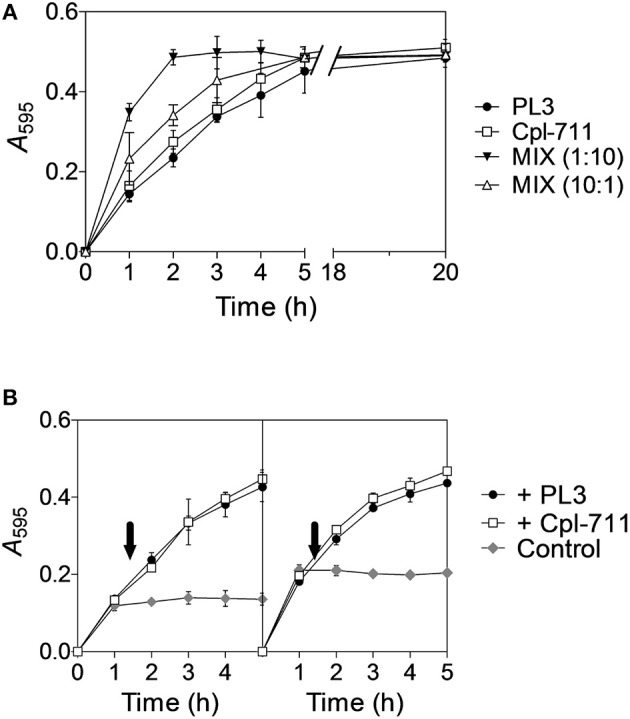
Synergistic degradation of *S. pneumoniae* R6 cell walls by Cpl-711 and PL3. **(A)** 1.3μM solution (≈50 μg/ml) of either PL3, Cpl-711, a mixture of both enzymes (MIX, either 1:10 or 10:1, respectively 0.13μM PL3 + 1.3μM Cpl-711, and 1.3μM PL3 + 0.13μM Cpl-711), or just PB as a no-treatment control and spectrophotometric blank read, were added onto pelleted RBB-stained R6 cell walls adjusting to A_595_ ≈ 0.5. Degradation products were measured spectrophotometrically after sample centrifugation at indicated time points. **(B)** RBB-stained cell walls were pre-treated with 1.3μM PL3 (Left panel) or Cpl-711 (Right panel) in PB for 1 h, and reactions were stopped by heat inactivation (80°C, 15 min). Incubation was then resumed at 37°C adding either 1.3μM PL3 (black circles), 1.3μM Cpl-711 (white squares) or an equivalent PB volume (gray diamonds) at the time indicated by the arrows.

To test the possible synergy between two different lysins we chose the most promising ones in terms of MIC and bactericidal activity, i.e., the lysozyme Cpl-711 and the NAM-amidase PL3. Initially, these two enzymes were tested using purified pneumococcal cell walls labeled with RBB and spectrophotometrically measuring the degradation products. This experiment showed that the PL3/Cpl-711 cocktail (in a molar ratio 1:10, 0.13μM PL3 plus 1.3μM Cpl-711) was able to degrade the cell walls in less than half the time it took for comparable concentrations (1.3μM) of PL3 or Cpl-711 alone (Figure 1A, reaction half-lives were 0.88, 2.07 and 2.05 h, respectively). When a small amount of Cpl-711 (0.13μM) was added together with 1.3μM PL3, an increase in reaction rate was also observed, though to a lesser extent (half-life = 1.47 h). In addition, the sequential enzymatic degradation of the substrate was tested. Cell walls were pre-treated with 1.3μM PL3 or Cpl-711 for 1 h. Afterwards, enzymes were heat-inactivated, and 1.3μM of either one enzyme or the other was supplied. The sequential treatment with different enzymes (i.e., addition of fresh Cpl-711 after pre-treatment with PL3 or vice versa) showed no significant difference with treating with the same enzyme in the two reaction steps, in terms of cell wall solubilization rates (Figure 1B).

**Figure 2:**
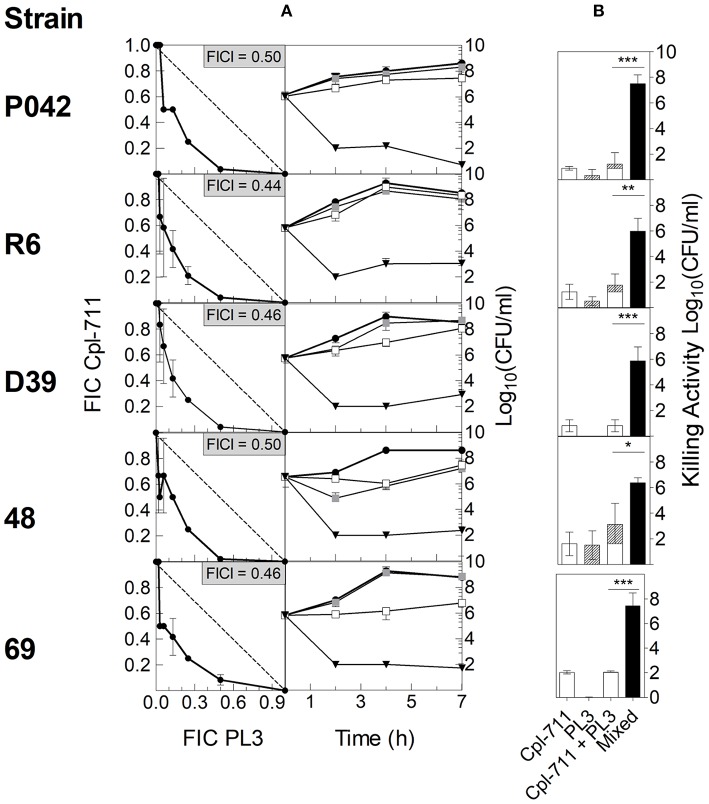
*In vitro* synergy testing of Cpl-711 and PL3 on pneumococcal cells. (**A**, left panel) Isobolograms of the checkerboard synergy testing method. In every panel, insets indicate FICI values for each strain. The dashed lines illustrate theoretical additive curves. (**A**, right panel) Time-killing assays treated with PB as control (black circles), with 0.25× MIC of Cpl-711 (white squares), with 0.25× MIC of PL3 (gray squares), or with a combination of 0.25× MIC of each enzyme (black triangles) for 7 h. Checkerboard and time-kill results are the results of at least three independent replicates. **(B)** Killing activity at 7 h of either 0.25× MIC of each enzyme added separately (Cpl-711, open bars; PL3, hatched bars) or 0.25× MIC of each enzyme added simultaneously (black bars). A statistical comparison by *t*-test was performed between the sum of the effects of the two enzymes added separately (third bar, which is the sum of the two previous bars) and the effect of the mixed enzymes treatment (fourth bar) (*P < 0.05; **P < 0.01; ***P < 0.001).

Then, the ability to synergistically inhibit bacterial growth *in vitro* was tested using checkerboard assays with strains R6, P042, D39, 48, and 69. The results for each strain were plotted in an *x/y* graph as isobolograms (Figure 2A, left panel). In all cases, the growth inhibition breakpoints depicted in the respective isobolograms describe a curve below the 135° line that stands for mere additive effect, indicating that the lysins do act synergistically. Confirming this point, the average FICI was in every case ≤ 0.5 (Figure 2A, left panel), which is the usual criterion for ascertaining synergy.

To complement these assays, time-killing experiments of such pneumococcal cultures, treated with subinhibitory concentrations of the two lysins (0.25× MIC of each lysin or 0.25× MIC of just one), were carried out. The comparison between the curves of single and combined lysin treatments on every strain further confirmed the existence of synergy between the two enzymes (Figure 2A, right panel), since the combined treatment provoked a dramatic decrease in bacterial viability when compared with the faint or even negligible effect of each enzyme alone. For example, MDR strain 69 (Figure 2A, right panel, fifth row) was not affected by 0.25× MIC PL3, and 0.25× MIC Cpl-711 was only able to inhibit growth up to 4 h incubation, after which, growth was resumed. In contrast, the combination of 0.25× MIC of each enzyme exerted a remarkable bactericidal effect from the very beginning, maintaining a low bacterial burden (10^2^ CFU/ml or below) during the whole experiment. For any of the strains tested, the decrease in cell viability with respect to the control at 7 h was ≥ 2 logs higher in the combined therapy than the sum of the effects of each enzyme used alone (Figure 2B).

Bacteriolytic activity of the enzyme combination was also observed under the microscope. Video S1 shows, in real time, the extremely fast lytic process of the combination of Cpl-711 and PL3 lysins on strain R6. The rapid and specific lysis of pneumococci was also evident in mixed cultures, where the same combination of lysins destroyed R6 pneumococci in a few seconds while keeping intact *S. aureus* CECT 86 cells, even when the incubation time was extended up to 15 min (Video S2).

### Synergistic Effect of Cpl-711 and PL3 on Biofilms

**Figure 3:**
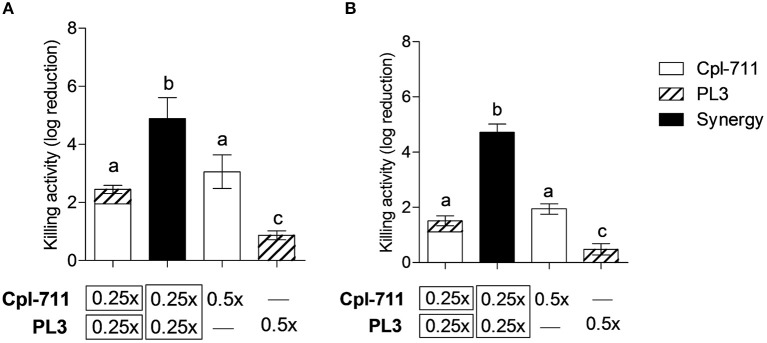
Synergistic bactericidal activity of Cpl-711/PL3 on *S. pneumoniae* P042 planktonic cells and biofilms. **(A)** Viable cells of planktonic (nonadherent) fraction of the cultures. **(B)** Viable cells of biofilms. Treatments with each enzyme, alone or in combination, were at the indicated concentrations (in times the MIC), according to the protocol explained in Materials and Methods. First bar represents the sum of the independent effects of adding either 0.25× MIC of Cpl-711 or 0.25× MIC of PL3, while second bar shows the effect of adding 0.25× MIC of Cpl-711 and 0.25× MIC of PL3 at the same time. Results are the mean ± SD of viable count differences with respect to the controls in log scale for at least three independent experiments. Log kills marked with the same letter are not significantly different from one another, whereas those with different letters are significantly different from one another, according to ANOVA followed with Tukey post-test (*P* < 0.05).

Bacterial biofilms pose an additional treatment challenge given their well-documented enhanced antimicrobial resistance. Nonetheless, lysins have been reported to have significant activity against *S. pneumoniae in vitro* biofilms (Domenech et al., 2011), although proper synergy was not tested according to conventional parameters. The combination of Cpl-711 and PL3 displayed a synergistic killing effect both in the planktonic fraction and in the adhered fraction of *S. pneumoniae* cultures, being the latter a more evident synergy (Figure 3). While on the planktonic cells the combination of 0.5× MIC of each enzyme causes a 2.4-log increase in killing activity, on the biofilm cells the synergistic effect increases by as much as 3.6 logs with respect to the sum of activities of the individual Cpl-711 or PL3 treatments.

### Synergy of Cpl-711 and PL3 on a Zebrafish Infection Model

**Figure 4:**
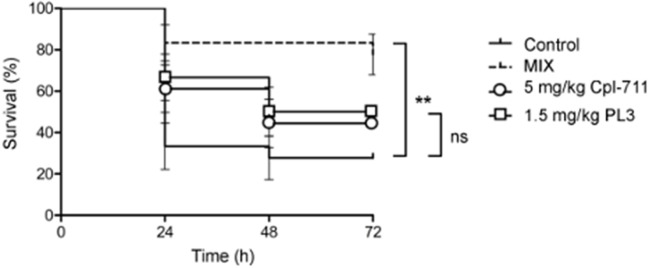
Zebrafish rescue from lethal pneumococcal infection by Cpl-711, PL3 and their combination (MIX). Survival of zebrafish infected with strain 48 and treated with PBS as control or 5 mg/kg of Cpl-711, 1.5 mg/kg of PL3, or a combination of half the concentration of each enzyme (MIX), monitored for a period of 72 h, is shown (**P < *0.01*; ns = non-significant difference).

Cpl-711 and PL3 combinations ranging from 0.5× to 2× MIC of each enzyme were initially tested on infection-free zebrafish embryos for acute toxicity. No signs of toxicity were detected after 72 h exposure to the antimicrobial compounds (100% survival with all concentrations of Cpl-711/PL3 combinations, ranging between 0.25 and 5 mg/kg, significantly different to the 41.7% survival of the 1 mg/ml paracetamol positive control). When adult zebrafish were infected with the MDR pneumococcal strain 48 only 27.8% of the control fish survived at 72 h after infection. After a treatment with 0.5× MIC of either Cpl-711 (5 mg/kg) or PL3 (1.5 mg/kg) 1 h post-infection, the survival percentage increased, respectively, to 44.4 and 50%. To test whether a synergistic effect could be observed *in vivo*, half the dose of each enzyme (i.e., 0.25× MIC Cpl-711 + 0.25× MIC PL3) was applied in a combined formulation. This corresponds to 3.25 mg/kg total enzyme (2.5 mg/kg Cpl-711 plus 0.75 mg/kg PL3). Under these conditions, the observed survival fraction at 72 h was 77.8%, clearly above to those of the individual lysins (Figure 4).

## Discussion

In recent years, phage-encoded lysins have demonstrated to be one of the most effective alternatives (*in vitro* and in animal models) for killing bacteria, including MDR strains. The possible therapeutic use of lysins would be focused against certain bacterial pathogens that pose high resistance levels against the common antibiotics of choice. In the case of *S. pneumoniae*, there are some capsular serotypes that cause most episodes of colonization and non-invasive disease of the respiratory tract as well as invasive infections, while evading vaccine protection and presenting high rates of resistance to common drugs.

In the pneumococcal system, Cpl-711 and PL3 lysins have displayed the most potent lethal activities, by hydrolyzing different peptidoglycan bonds. Although both enzymes are CBPs (with CBDs from different origins), indeed they comprise catalytically distinct murein hydrolase domains: Cpl-711 bears the *Glyco_hydro_25* lysozyme domain (Pfam PF01183) from Cpl-7 and the CBD from Cpl-1. Both Cpl-7 and Cpl-1 have catalytic domains from the same family (*Glyco_hydro_25*), sharing between them all but 27 residues (with a 95.2% similarity) (Díez-Martínez et al., 2015). Still, the chimera is more potent than any parental enzyme, Cpl-1 included, as our MIC results point out. On the other hand, PL3 was constructed with the Pal *Amidase_5* domain (Pfam PF05382), fused to the CBD of LytA. Our results also confirm that PL3 has a superior ability to control pneumococcal growth when compared with its parental enzyme. Still, PL3 has a quite lower MIC value than Cpl-711, and this difference also appears when comparing the most potent parental proteins (Cpl-1 and LytA) between them. Both our results here and previous data suggest that Cpl-711 and PL3 are in fact very similar in terms of *in vitro* catalytic degradation ability and they both display a high bactericidal activity (Díez-Martínez et al., 2015; Blázquez et al., 2016). Hence, the observed differences in growth inhibition capacity might be related to features other than catalytic degradation or bactericidal activity, such as long-term protein stability in complex media or interaction with the bacterial surface. Such characteristics could be related to the LytA and PL3 common CWB repeats, different in sequence than those of Cpl-711 or Cpl-1 although all of them bind phosphocholine residues.

Regarding the behavior of the Cpl-711/PL3 combined treatment, the main effect observed in cell wall degradation experiments was an increase in the reaction velocity when using the combination of Cpl-711 and PL3 with respect to each enzyme alone. This is to say that the ability of the mixture to hydrolyze two different bonds within the peptidoglycan allows a faster solubilization of cell wall fragments (and then, putatively, a chance to faster damage pneumococcal cell wall to a point that osmotic pressure causes cell death). It has been described for other polysaccharide degrading enzymes (i.e., some cellulases) that their synergistic activity when they break distinct linkages on the polymeric mesh is due to a sequential double action (Kostylev and Wilson, 2014). In such example, a single initial treatment with one of the synergistic pair is able to “erode” the surface of crystalline cellulose, creating new sites (chain ends) for the second enzyme to act, as well as uncompressing the initially tight cellulose network facilitating its access. However, it does not seem to be an identical case for lysins (or at least these lysins in this particular bacterial system), since the sequential addition (in any order) of Cpl-711 and PL3 did not produce the synergistic reaction velocity increase that was observed when these enzymes were used simultaneously. Another interesting point to raise is that the best synergistic Cpl-711/PL3 degradation rate increase was observed when just a comparatively small amount of PL3 was added to the reaction mixture. However, adding a small amount of Cpl-711 together with a greater amount of PL3 did not result in such a dramatic degradation rate increase. We speculate then that NAM-amidase peptidoglycan bonds cut by PL3 might facilitate Cpl-711 action by granting a better access to its substrate, or a better basis for further peptidoglycan destructuring by Cpl-711 lysozyme activity. This facilitation, however, must continue all along the degradation process for it to be truly synergistic, probably because of the homogeneous entanglement along the whole peptidoglycan mesh of the two kinds of targeted bonds. We envision the model as if PL3 would “clear the path” for a better Cpl-711 activity toward peptidoglycan degradation, layer by layer, within the peptidoglycan polymer. Additional evidence, however, would be necessary to confirm such a mode of action.

When the Cpl-711/PL3 combination was employed on actively growing pneumococcal cultures, a synergistic increase appeared, measured either as growth inhibition or as bactericidal effect. This means an outstanding killing efficiency, greater than in any prior antipneumococcal synergy reports, with respect to the total enzyme mass employed. The different strains tested allow to discard any interference of such features as the presence of autolysins or a capsule in the synergistic effect, since the results depicted here do not show great differences between R6, P042, and D39. These are isogenic strains that only differ in: the lack of LytA and LytC autolysins (P042), or the presence (D39) or absence (R6, P042) of capsule. The additional MDR strains assayed suggest that such synergy could be extrapolated to other clinical strains such as the tested *S. pneumoniae* 48 and 69. The bactericidal activity of Cpl-711 and PL3, and their synergistic effect, have also been demonstrated against pneumococci growing in biofilms. The ability to eliminate and kill the cells forming a bacterial biofilm has been reported for several lysins, including antipneumococcal ones (Domenech et al., 2011). In this case, the stress is put on the great death rate caused in the P042 pneumococcal cells of the biofilm (3.6 logs of killing greater than the sum of the individual lysins treatments in just 1 h of incubation). The synergistic effect was more appreciable in the biofilm fraction than in the planktonic one. This characteristic has been pointed out before, since the increased activity reported for the LytA/Cpl-1 combination, higher than the sum of effects of the individual treatments, could only be detected in a biofilm model (Domenech et al., 2011), but not by the checkerboard assay (Rodríguez-Cerrato et al., 2007a). This might be due to the particular setting of the biofilm, which differentiates from the planktonic mode of growth basically in two aspects: (a) the metabolic and regulatory shift that occurs upon biofilm lifestyle adaptation; and (b) the sessile interconnectedness of biofilm cells, which become surrounded by an extracellular matrix that contains proteins, DNA, and polysaccharides.

In addition to all these *in vitro* experiments, the synergistic effect of Cpl-711 and PL3 has been confirmed in an adult zebrafish infection model. This has proved to be an appropriate model to study bacterial infections, with several practical advantages over the usual murine models (Torraca and Mostowy, 2018), as well as showing a good correlation in the results obtained in such mice models (Letrado et al., 2018). The bactericidal activity of several lysins has been previously validated on zebrafish embryos (Díez-Martínez et al., 2015; Blázquez et al., 2016), but in this work adult zebrafish, which present an adaptive immune response that is absent in the embryos, making them more relatable to the infection setting in higher animals such as humans, were treated by microinjection both of the bacterial challenge and the lysins. The positive result supports the possibility of successful application of Cpl-711/PL3 in real *in vivo* infection settings, although more experimental evidence must be accumulated.

One of the possible concerns about lysins, when applied *in vivo* as therapeutic agents, is their short half-lives in blood, e.g., 20–22 min for Cpl-1 and LytA, in murine models (Loeffler et al., 2003; Rodríguez-Cerrato et al., 2007b). Neverthelees, the great killing speed of these enzymes, enhanced in the Cpl-711/PL3 combination, as showed in Figure 2A (right panel) or Video S1, together with the extensive evidence of *in vivo* efficacy, lessens this concern. Besides this rapidity in provoking cell death upon contact, Video S2 also illustrates a characteristic feature of CBP-lysins, which is their exquisite selectivity for those bacteria bearing phosphocholine residues in their teichoic acids, namely pneumococcus and a few other respiratory tract-resident bacteria such as *Streptococcus mitis* or *Streptococcus oralis* (Díez-Martínez et al., 2015; Blázquez et al., 2016). This behavior foresees the application of these lysins to selectively kill *S. pneumoniae* leaving mostly intact the rest of the microbiota.

## Conclusion

A synergistic effect of Cpl-711 and PL3 lysins to effectively kill pneumococcal strains has been demonstrated. This bactericidal activity is evident both on planktonic cells as growing in biofilm, and it was confirmed in an *in vivo* model of infection. The increasing evidence, to which this work adds, on synergy between lysins cleaving different bonds in the peptidoglycan mesh, encourages the search for catalytically diverse enzymes. Hence, the results presented here imply the possibility to formulate highly efficient therapies based on the engineered enzymes combination that could improve the ability of lysin therapy to eradicate infectious bacteria in a specific, safe and rapid manner, and thus envisions a future application of such therapies to overcome the antibiotic-era exhaustion.

## Data Availability

The datasets generated for this study are available on request to the corresponding author.

## Ethics Statement

Animal experiments were conducted at Ikan Biotech SL in The Zebrafish Lab department and performed according to European Union guidelines for handling of laboratory animals (http://ec.europa.eu/environment/chemicals/lab_animals/home_en.htm). Approval for these studies was granted by the University of Navarra's Ethics Committee for Animal Experimentation (Protocol 034-17).

## Author Contributions

RV and PG designed the experiments, which were performed by RV except for the *in vivo* model. RV and PG discussed the results and wrote the manuscript. Both authors read, edited, and approved the final manuscript.

### Conflict of Interest Statement

The authors declare that the research was conducted in the absence of any commercial or financial relationships that could be construed as a potential conflict of interest.

## Abbreviations

CBD: choline binding domain
CBP: choline-binding protein
CWBD: cell wall-binding domain
FICI: fractional inhibitory concentration index
MDR: multidrug resistant
RBB: Remazol Brilliant Blue.
