# Supplementary material for: Synergy Between Two Chimeric Lysins to Kill Streptococcus pneumoniae
Source: Front Microbiol. 2019 Jun 5;10:1251. doi: 10.3389/fmicb.2019.01251 (PMC6560164; doi:10.3389/fmicb.2019.01251)
Supplement: Supplementary file 1 [file Data_Sheet_1.PDF]

SUPPLEMENTARY MATERIAL

Synergy between Two Chimeric Lysins to Kill *Streptococcus pneumoniae*

Roberto Vázquez[1,2] and Pedro García[1,2*]

[1]Departamento de Biotecnología Microbiana y de Plantas, Centro de Investigaciones Biológicas, CSIC, Madrid, Spain, [2] CIBER de Enfermedades Respiratorias, Madrid, Spain

Running title: Synergy between lysins to kill pneumococci

[*]***Correspondence:*** *Pedro García (pgarcia@cib.csic.es)*

Specialty section: *Antimicrobials, Resistance and Chemotherapy*

Contents:

Table S1

Figure S1

Video S1

Video S2

TABLE S1 | *E. coli* strains and plasmids used for antipneumococcal enzybiotics production.

Strain	Plasmid	Protein	Reference
BL21 (DE3)	pMMN1	LytA	(Moscoso et al., 2010)
DH5α	pMSP11	Pal	(Sheehan et al., 1997)
BL21 (DE3)	pET29-PL3	PL3	(Blázquez et al., 2016)
RB791	pCP700	Cpl-7	(Díaz et al., 1991)
DH5α	pCIP100	Cpl-1	(Sanz et al., 1992)
BL21 (DE3)	pTRD762	Cpl-711	(Díez-Martínez et al., 2015)

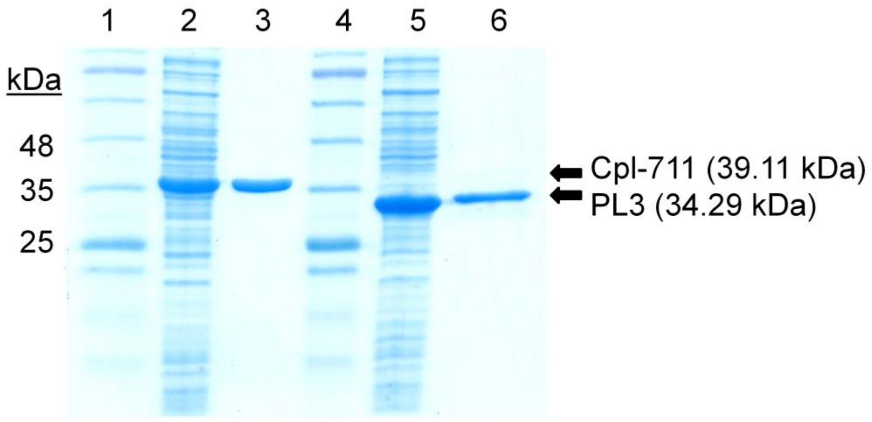

FIGURE S1 | SDS-PAGE of the production and purification of the chimeric lysins Cpl-711 and PL3. Lanes 1 and 4: molecular weight markers (NZYColour II Protein Marker, NZYTech); lanes 2 and 5: crude soluble extracts from induced *E. coli* BL21(DE3) cells with recombinant plasmids producing, respectively, Cpl-711 and PL3; lanes 3 and 6: Cpl-711 and PL3 proteins, respectively, from DEAE-Sepharose columns and purified in a single step.

VIDEOS S1 and S2 | Bacteriolytic effect of the combination of enzymes (Cpl-711 and PL3) observed under the microscope. Exponential cultures of either the pneumococcal P042 strain alone (Video S1), or with *S. aureus* CECT 86 strain (mixed at the same $OD_{550} \approx 0.3$) (Video S2), were centrifuged and the pellet washed twice with distilled water. The cells were then resuspended in distilled water and adjusted to $OD_{550} \approx 0.6$. A sample of both bacterial suspensions was spread onto a microscope slide and let air-dry. Finally, 5 µl of a solution containing the final concentration of the enzymes were added, at a final combination of 10× MIC each, and observed with a Leica DM4000B microscope under a 100× oil objective. Recording started immediately after the enzyme addition, using an iPhone 6S with an adapter for mounting it onto the ocular lens. Videos are shown in real time.

REFERENCES

Blázquez, B., Fresco-Taboada, A., Iglesias-Bexiga, M., Menéndez, M., and García, P. (2016). PL3 amidase, a tailor-made lysin constructed by domain shuffling with potent killing activity against pneumococci and related species. *Front. Microbiol.* 7, 1156. https://doi.org/10.3389/fmicb.2016.01156.

Díaz, E., López, R., and García, J. L. (1991). Chimeric pneumococcal cell wall lytic enzymes reveal important physiological and evolutionary traits. *J. Biol. Chem.* 266, 5464–5471.

Díez-Martínez, R., De Paz, H. D., García-Fernández, E., Bustamante, N., Euler, C. W., Fischetti, V. A., et al. (2015). A novel chimeric phage lysin with high *in vitro* and *in vivo* bactericidal activity against *Streptococcus pneumoniae*. *J. Antimicrob. Chemother.* 70, 1763–1773. https://doi.org/10.1093/jac/dkv038.

Moscoso, M., Domenech, M., and García, E. (2010). Vancomycin tolerance in clinical and laboratory *Streptococcus pneumoniae* isolates depends on reduced enzyme activity of the major LytA autolysin or cooperation between CiaH histidine kinase and capsular polysaccharide. *Mol. Microbiol.* 77, 1052–1064. https://doi.org/10.1111/j.1365-2958.2010.07271.

Sanz, J. M., Díaz, E., and García, J. L. (1992). Studies on the structure and function of the N-terminal domain of the pneumococcal murein hydrolases. *Mol. Microbiol.* 6, 921–931.

Sheehan, M. M., García, J. L., López, R., and García, P. (1997). The lytic enzyme of the pneumococcal phage Dp-1: a chimeric lysin of intergeneric origin. *Mol. Microbiol.* 25, 717–725.
